# The Teeth a Test of Age

**Published:** 1838-01

**Authors:** 


### Art. XX.

*The Teeth a Test of Age, considered with Reference to the Factory Children. Addressed to the Members of both Houses of Parliament.* By Edwin Saunders.—*London*, 1837. 8vo. pp. 76.

This is a clever little pamphlet upon a very important subject. Of all the means hitherto proposed for ascertaining the age of children, there is not one, taken by itself, but in a great proportion of cases would be useless; and accordingly we find that deception has been practised to a great extent, in the manufacturing districts, both by the employers and the employed, and the recent benevolent parliamentary enactments thereby evaded. The interval allowed by these enactments, between the age of helpless infancy and that when hard and continuous labour is to commence, is short enough; yet, even this small period, which is all that can be looked to for training the children of the manufacturing districts to such habits, tastes, and feelings as may render them honest, industrious, intelligent, and happy, is neglected and encroached upon. We know from eye-witnesses that children are even now, after all that has been said and written on the subject, doomed to work at an age when their physical development proves them to be incapable; and the premature deaths of thousands of these unhappy beings demonstrate what must of necessity be the consequence. There is but one remedy which would strike at the root of the evil at once, and that is a system of national education, similar in some respects to that which is now in operation in some parts of the continent. This, with the present mode of registration, would not only ensure a sufficient time for the development of the body, but would convert what is now a mere automaton into a thinking, reasonable being. In the mean time, it is important to examine every hint which is thrown out towards rendering the end in view more certain; and we think the observations of Mr. Saunders, as he himself says, "cannot be unworthy the attention of the legislature, in its humane endeavours to succour and protect 35,000 little sufferers from injustice and oppression."

The question to be decided is, whether the Teeth obey certain fixed laws in their progressive eruption, so as to render the age of an individual, up to a certain period, ascertainable from an inspection of them?

"An acquaintance with the manner of their production, their structure, and the mode and order of their eruption, would certainly not discourage the suggestion: on the contrary, their great density of structure, unique and nearly independent mode of formation, together with their almost total want of sympathy with those constitutional changes and variations in the health in which the more highly organized structures partake, would induce the expectation of a greater uniformity of development of the teeth than of any other parts of the frame. And the idea would receive additional confirmation from the well-known fact that, amongst the lower animals, by common consent, it is regarded as the most certain and constant of all proofs on the subject that can be obtained: it is, therefore, not unreasonable to suppose that, making adequate deductions on account of the interruptions to normality of development, which a more artificial mode of existence opposes, the same regularity would obtain in the human species." (P. 27.)

It would be natural to suppose, *a priori*, that diseases affecting the development of the body would also, in some degree, affect that of the teeth; but the author assures us this is not the case, or at least not to such an extent as materially to affect the correctness of the results. He contends that the formative process is never entirely stopped by causes acting on other portions of the animal economy; that disease may exist in one part, retard the full development of the physical powers, but that still the teeth, as if belonging to another and separate system, are equally well developed as in cases where all the functions are normal; that, in fact, they are developed according to a law of their own. It will be seen from this that the test cannot supersede the other means used for the same purpose,—viz. the ascertaining the state of health, constitution, temperament, &c. These alone can give any indication of physical strength. The only point gained is the knowledge of certain signs connected with dentition, which afford a criterion of age.

It is only necessary, according to the present Factories' Bill, to ascertain two epochs,—viz. nine and thirteen years of age; and accordingly the author has confined himself, in his investigations, to these periods, although tables could be formed equally applicable to the other periods.

The following is an instance showing the superiority of this test.

| | HEIGHT. | | AGE. | | INCISOR TEETH. | | MOLAR TEETH. |
|---|---|---|---|---|---|---|---|
| | Feet. | Inches. | Years. | Months. | Central. | Lateral. | Anterior. |
| James Jacques...... | 4 | 0¾ | 8 | 4 | 3 | 0 | 4 |
| John Sims .......... | 3 | 7¼ | 8 | 7 | 3 | 2 | 4 |

"Here, then, was a difference of *five inches and a half* in height in favour of the youngest boy, who was by *three months* the junior of the other. It need scarcely be observed, that the elder and shorter boy was by far the strongest and most capable of exertion: his pulse was strong and regular, while that of the latter child was small and frequent, indicating a low degree of animal power. Judging, then, from the height in this case, the elder boy, who was active and shrewd, and displayed great physical and mental energy, would have been pronounced too young for labour; while the other, *who was junior by three months*, but who exceeded him in stature by *five inches and a half*, would have been subjected to a distressing and injurious amount of exertion. It will be seen, however, that the appearances of the mouth indicated the true state of the case; so that, relying upon these, the shorter child must have been the elder." (P. 54.)

The author thinks he is justified in laying it down as a law, that both teeth are to be considered as emerged from the gums, wherever the tooth on one side is freely developed. It is necessary to direct attention to this, since it will make a great difference in the results, if unattended to.

Upwards of 1000 children have been examined by Mr. Saunders in the various large schools in London, and elaborate tables show the dental development at the two epochs; the results are summed up in the following manner:

"Thus, then, it appears that, of 708 children of nine years of age, 389 would have been pronounced, on an application of this test, to be near the completion of their ninth year; that is, they presented the full developments of that age. But, on the principle already stated, that of reckoning the fourth teeth as present where the three are fully developed, a still larger majority will be obtained, and, instead of 389, the proportion will be as follows:—Of 708 children, no less a number than 530 will be fully nine years of age. What, then, are the deviations exhibited by the remaining 178? They are the following:—126 would be pronounced eight years and six months, and the remaining 52 eight years of age; so that the extreme deviations are only twelve months, and these only in the inconsiderable proportion (when compared with the results obtained by other criteria,) of 52 in 708.

"Again, of 338 children of thirteen years of age, no less than 294 might have been pronounced, with confidence, to be of that age. The remaining 44 would have been considered as follows:—Thirty-six in their thirteenth, and eight near the completion of their twelfth year." (P. 76.)

Such is an outline of the evidence adduced by Mr. Saunders; and it is, in our opinion, quite sufficient to establish the superiority of the dental test over every other yet proposed. The subject is well worthy the consideration, not only of the government, as connected with the Factory Bill, but also of the medical jurist, to whom tables such as those drawn up by the author would, in many cases, prove of essential service.

---

